# Response to Letter to the Editor, Re: The Effect of Nirmatrelvir-Ritonavir on Short- and Long-term Adverse Outcomes From COVID-19 Among Patients With Kidney Disease: A Propensity Score-Matched Study Open Forum Infect Dis. 2024 Dec 31;12(1):ofae756. doi: 10.1093/ofid/ofae756

**DOI:** 10.1093/ofid/ofaf481

**Published:** 2025-08-21

**Authors:** Ian Strohbehn, Meghan Sise

**Affiliations:** Department of Nephrology, Massachusetts General Hospital, Boston, Massachusetts, USA; Department of Nephrology, Massachusetts General Hospital, Boston, Massachusetts, USA

In response to Dr Rubin, we note that 572 patients (52%) received nirmatrelvir 300 mg/ritonavir 100 mg twice daily for 5 days and 523 patients (48%) received nirmatrelvir 150 mg/ritonavir 100 mg twice daily for 5 days. Since baseline glomerular filtration rate (GFR) was defined in our study using the median of all creatinine values in the 365 days prior to infection calculated using the CKD-EPI 2021 equation, it is possible that prescribing doctors may not have had access to a recent GFR, or may have selected the dose based on the most recent GFR result that was close to or above 60 mL/min/1.73m². Additionally, some physicians and pharmacists may have recommended a dose based on Cockcroft Gault Creatinine Clearance. Since 74% of the matched antiviral-treated patients fell into the GFR 45–60 range, alternative GFR estimates could have led to selection of full-dose treatment in numerous patients in our cohort. It is also possible that some physicians were not aware of the recommended dose reduction early after the emergency use authorization of nirmatrelvir/ritonavir. In terms of outcome differences between full and reduced doses of nirmatrelvir–ritonavir, there were no significant differences in any of the short or long-term adverse outcomes, including hospitalization within 30 days (1.4% in full dose and 1.3% in reduced dose, *P* = 1.0) and death within 365 days (0.8% in full dose and 1.4% in reduced dose, *P* = .21).

